# Tracking volcanic explosions using Shannon entropy at Volcán de Colima

**DOI:** 10.1038/s41598-023-36964-x

**Published:** 2023-06-17

**Authors:** Pablo Rey-Devesa, Janire Prudencio, Carmen Benítez, Mauricio Bretón, Imelda Plasencia, Zoraida León, Félix Ortigosa, Ligdamis Gutiérrez, Raúl Arámbula-Mendoza, Jesús M. Ibáñez

**Affiliations:** 1 Department of Theoretical Physics and Cosmos, Science Faculty, University of Granada, Avd. Fuentenueva s/n, 18071 Granada, Spain; 2 Andalusian Institute of Geophysics, Campus de Cartuja, University of Granada, C/Profesor Clavera 12, 18071 Granada, Spain; 3 Department of Signal Theory, Telematics and Communication, Informatics and Telecommunication School, University of Granada, 18071 Granada, Spain; 4 Centro Universitario de Estudios Vulcanológicos (CUEV), Observatorio Vulcanológico, Universidad de Colima, Colima, Mexico

**Keywords:** Geophysics, Seismology, Volcanology, Mathematics and computing

## Abstract

The main objective of this work is to show that Shannon Entropy (SE) calculated on continuous seismic signals can be used in a volcanic eruption monitoring system. We analysed three years of volcanic activity of Volcán de Colima, México, recorded between January 2015 and May 2017. This period includes two large explosions, with pyroclastic and lava flows, and intense activity of less energetic explosion, culminating with a period of quiescence. In order to confirm the success of our results, we used images of the Visual Monitoring system of Colima Volcano Observatory. Another of the objectives of this work is to show how the decrease in SE values can be used to track minor explosive activity, helping Machine Learning algorithms to work more efficiently in the complex problem of distinguishing the explosion signals in the seismograms. We show that the two big eruptions selected were forecasted successfully (6 and 2 days respectively) using the decay of SE. We conclude that SE could be used as a complementary tool in seismic volcano monitoring, showing its successful behaviour prior to energetic eruptions, giving time enough to alert the population and prepare for the consequences of an imminent and well predicted moment of the eruption.

## Introduction

One of the great challengers in vulcanology is studying the behaviour of volcanoes during eruptive episodes, in order to understand the underlying physical processes and to develop warning systems to minimize those risks^1–3^**.** Volcanic eruptions involve highly energetic interactions between the inner fluid dynamic and the medium. Therefore, its understanding embraces the use of several disciplines such as geochemistry, geology or geophysics^4–6^**.** With this kind of studies, volcanologists are advancing successfully in the development of forecasting tools to predict eruptive episodes in the last decades. However, the high variety of volcanic scenarios and eruptive styles when an eruption could happen make forecasting a complex problem to solve. Each volcano is a different system itself, with different source mechanisms and various eruptive dynamics. Therefore, at this time there is not a true universal way to board the pre-eruptive activity making the prediction of an eruption a difficult task. One of the most successful tools when forecasting an eruption is the use of seismic data ^7–10^**.** Volcanic activity associated to magma movement or gas emission, generates seismicity that can be recorded through time as a seismic signal. Seismicity has been used in different ways for the development of early warning tools. Based on the type of signal, its frequency content, duration, energy, spatial position within the volcano and many other parameters, it is possible to make precursor eruption models, some with evident success^9,11,12^**.** In general, the study of the energy released, and some models derived from it, has been one of the most widely used tools^13–17^.

Machine Learning (ML) techniques allow better identification of events and greater completeness of databases^18–22^**.** However, even though these methods are widely adopted around volcanic observatories, there are several topics that still lack of a strong solution, as transferability to other volcanic systems^17,23–25^. Moreover, even the same volcano may erupt in different ways (closed vent or open vent, depth of the reservoir, energy accumulated, etc.) and different processes may occur at the same time^26–28^**.** Thus, the big amount of labelled database required usually obstructs the development of a simple, reliable and exportable system. Seismic records may exhibit increasements of the energy that scientists use to forecast eruptive episodes. Through both energy-based methods and automatic earthquake classification systems, vulcanologist have achieved numerous forecasting successes. However, the uniqueness of every volcano and the variety of its type of eruptions makes of these methods non-universal tools.

Rey-Devesa et al.^29^ proposed a promising early-warning tool, tested in different volcanic systems (Bezymianny, Mt. Etna, Kilauea, Augustine and Mount St. Helens) and in different eruptive episodes of the same volcano. This approach applies advanced signal analysis techniques to extract a set of underlying parameters of the seismic signal and study their temporal evolution. These authors developed a short-term volcanic early-warning tool working efficiently and successfully in these scenarios. They observed how the decrease in Shannon Entropy (SE) generated stable pre-eruptive signs from around 5 days prior to a large explosion, to tens of hours in the case of lava fountains.

In this work we will advance in this line showing the SE can be used routinely in a seismic monitoring system in a reliable way. We study a long time period of seismic record in an active volcano with a wide variety of eruptive processes. We analyse three years of intense volcanic activity of Volcán de Fuego de Colima, (2015–2017). This analysis includes at least two large volcanic explosions, two pyroclastic flows, an effusive period, less energetic explosions, and a period of quiescence that is lasting until at least the date of the present work^30,31^. The analysis of the quiescence stage is important because it can help us demonstrate that SE variations appear as one-to-one and stable indicators of a pre-eruptive alert, i.e., SE is differentiable^17^. Therefore, its implementation in volcanic surveillance systems could be crucial to determining a possible reactivation of this volcanic system. The seismic analysis is complemented with the images of the Visual Monitoring system that the Colima Volcano Observatory has, obtaining confirmation of how the eruptive episode was. We were able to visually confirm the volcanic origin of more than 70% of the SE minima considered as potential eruptive episodes. In the remaining 30% of the potential false cases night and clouds affected to not being able to identify volcanic activity in the visual records.

We also show how the decreases in the SE can also be used as a seismic alternative to track this minor explosive activity, and help ML algorithms. Some volcano-seismic event classification systems based on ML approaches used in forecasting involve a category associated with volcanic explosions, debris flow and effusive eruptions^32^.

## Volcanic framework and data

**Figure 1:**
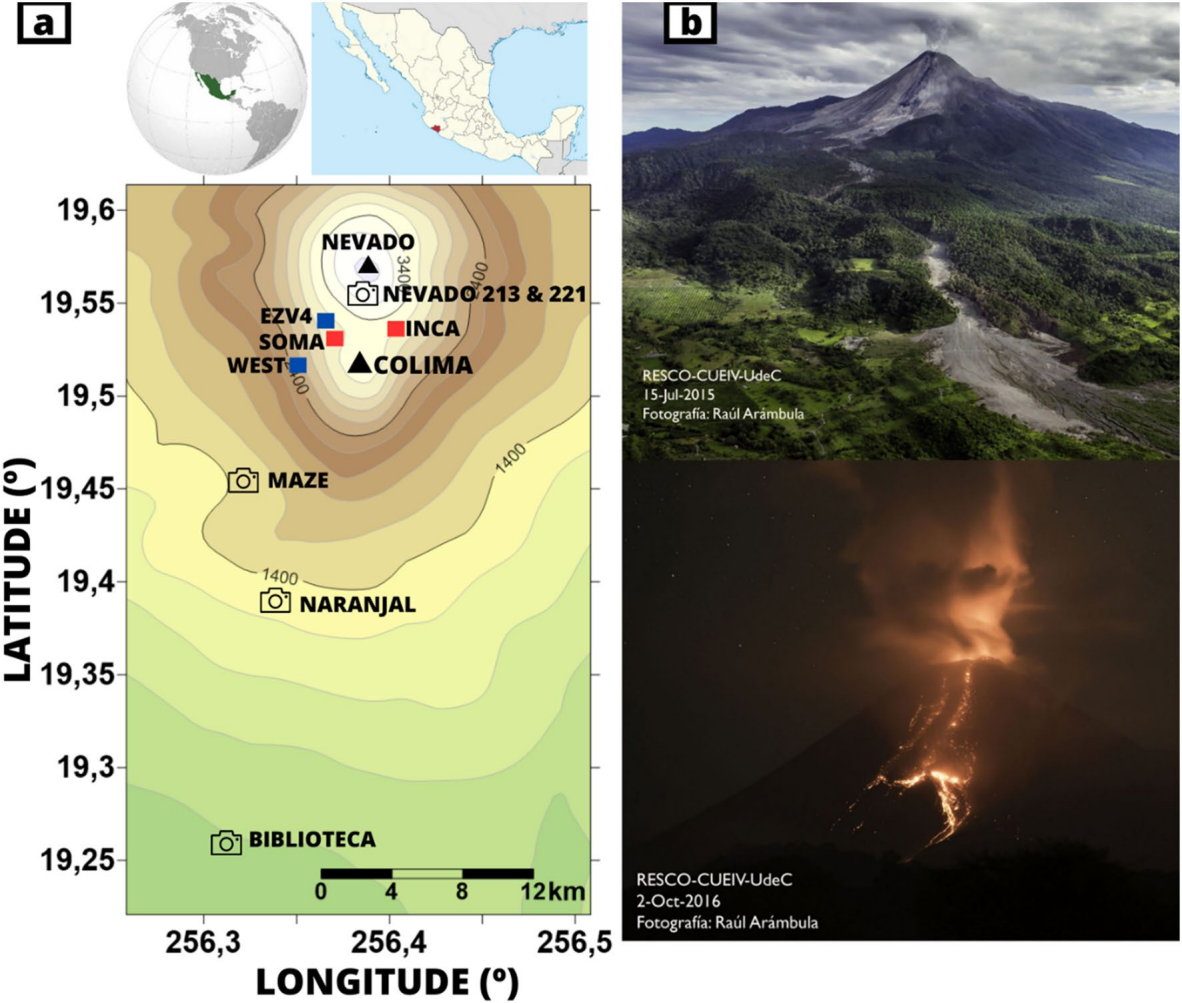
(**a**) Map of the seismic stations (squares) and visual cameras (cameras) monitoring Volcán de Colima, Mexico. Black triangles show the old Nevado volcano and the active Volcán de Colima. Red squares are the representing seismic stations in this work. Map made with Surfer 16 (https://www.goldensoftware.com/products/surfer). (**b**) Pictures of eruptive episodes analyzed; from upper to lower: path of the pyroclastic flow occurred on July 11th 2015; vulcanian eruption started on October 1st 2016. Pictures of Raúl Arámbula-Mendoza.

Volcán de Colima is an andesitic stratovolcano located in western Mexico (Fig. 1) and represents one of the most studied volcanoes in the world due to its high activity, being considered as one of the most active of the North American continent. There are evidences of its very high explosive activity since the beginning of the sixteenth century^33,34^. This volcano is able to have very intense volcanic activity after period of tens of years of quite state including very energetic activity as the growing of El Volcancito lateral vent^35^. In 1913 a Plinian eruption destroyed the summit of the volcano and generated various pyroclastic flows; a similar eruption nowadays would affect around half a million people^36^ turning Volcán de Colima into a very hazardous volcano. Volcán de Colima is being monitored since 1989, recording many periods of moderate effusive activity and processes like dome building and destruction, lava flows, pyroclastic flows, or vulcanian eruptions^37–39^. Volcanic activity is characterized by eruptive cycles between which the volcano does not show any type of activity, but its petrological characteristics explain the explosive nature of its main eruptions^40,41^. The reactivation of the volcanic system starts with phases of dome-growing at the summit zone^42^; after that, lava and pyroclastic flows are emitted^43^, followed by frequent explosions of variable intensity^44–46^. The cycle uses to end with a plinian eruption that destroys the summit region.

After signs of reactivation in January 2013, Volcán de Colima gradually increased its effusive and explosive activity, including the growing of a dome in the summit area. Among the different eruptive episodes, in this manuscript we will pay our attention in the analysis in detail of three different periods:

- On July 10th–11th, 2015 Volcán de Colima erupted producing 2 pyroclastic flows (named also Pyroclastic Density Currents) and destroying the summit dome^30,47^. The episode was the most energetic since 1913 Plinian eruption. Prior to this eruptive episode, only few volcano-tectonic events, usually considered as an important precursor, where detected^48^. This implies that a classic forecasting strategy based on the increasing number of earthquakes and their evolution from VTs to other events^9,11^ does not fit for this type of eruptions. Therefore, this is the ideal scenario to test how reliable is our approach of forecasting using the SE. This eruption was preceded by an increasing number of rockfalls and degassing activity, with elevated fumarolic activity and SO_2_ flux^30^. On July 10th at 20:16 h, the collapse of the dome produced a first pyroclastic flow that reached 9.1 km in length and lasted 52 min. Around 16 h later a second event occurred, lasting 1 h and 47 min and reaching 10.3 km in length.
- The second episode is an effusive volcanic activity occurred between 26 of September and 1 of October of 2016. The lava dome overflowed the crater rim and sent a slow-moving lava flow that reached more than 2 km in length and occasional pyroclastic flows^49^. This episode finalized with a moderate volcanic explosion but no important pre-eruptive seismicity was recorded.
- Explosive stage during January–February 2017 with a set of moderate volcanic explosions culminating with an explosion whose ash cloud reached up 5 km over the submit crater occurred the 3 of February of 2017, followed by minor eruptive episodes. After this moment no seismicity neither other external volcanic activity is measured in the volcano with the exception of moderate fumarolic emission and thermal anomalies.

We analysed continuous seismic record of a set of seismic stations belonging to the Telemetric Seismic Network of Colima (RESCO). RESCO is a part of the Centre for Studies and Volcanological Research (CUEV) of the University of Colima, and manage the monitoring of the volcano. The seismic network used in this analysis has 4 short-period SS-1 Ranger vertical seismometers and 6 broadband Guralp CMG-40TD and CMG-6TD, with a sampling rate of 100 Hz ^31^**.** In this work we used data from the stations SOMA, WEST, INCA and EZV4 (Fig. 1) recorded between January 2015 and March 2017. In this manuscript we will show results obtained for stations SOMA and INCA due to their temporal completeness. SOMA and INCA are the closest stations to the crater, located at less than 2 km.

In addition, to cross check our seismic results with evidences of the explosive episodes we used photos and videos of the volcanic activity. Volcán de Colima is video monitored in real time with a network of several stations that transmit images continuously to the observatory in the CUEV^50,51^. We used images from cameras: Biblioteca, Cuauhtemoc, MAZE, Naranjal, Nevado 213 and Nevado 221.

## Method

SE is a statistical feature associated to the waveform and its frequency content. We take the vertical component of the raw seismic signal and use a bandpass filter to filter the signal between 1 and 16 Hz. We selected this frequency band to avoid low and high frequency noise associated to climatic conditions like wind or storms. Then we create a moving window overlapped along the seismic temporal vector. The length of this window varies in function of the target of our search and the length of the period analysed. For the systematic analysis of the almost three years of seismic record, we used a window length of 10 min. For the study of the largest explosions, the window length was of 10 min. In the case of low energy eruptive episodes, the briefness of the changes led us to use shorter windows of 1 min. In all cases the used windows were overlapped a 50%. We calculated the SE in the frequency domain in every window and then we built a vector with the temporary evolution of the entropy. The Eq. 1 show the expression used to calculate the SE^52,53^:

1 $$SE=-{\sum }_{i}P\left({S}_{i}\right){log}_{2}\left(P\left({S}_{i}\right)\right)$$

where $P\left({S}_{i}\right)$ is the probability density function of the seismic record, on the frequency domain.

According to Eq. (1), since the analysis is done in the frequency domain, SE is associated to the homogeneous frequency contains of the signal. When seismograms are composed by random signals (e.g., background or cultural noise), or by a set of non-homogeneous volcanic signals, then values of SE are high. In case of the occurrence of a continuous arrival of homogeneous signals with same or coherent frequency content, then SE must have lower values, since the probability to find similar signals moves toward 1, and log of 1 is zero. The main advantage of this parameter compared to other methods is that this SE excursion to zero is independent of the type of recorded seismicity and its energy. For example, if the pre-eruptive seismicity is composed of VT earthquakes (dominated by high frequencies) the SE will move to zero since VTs dominate over the rest of seismicity and P(Si) will be moved toward 1. This behavior will be the same if the pre-eruptive signal is a volcanic tremor (dominated by low frequencies), or a mix of seismicity. The necessary condition for SE to move towards zero is that the seismic signal is homogeneous, in the frequency domain, over time. Therefore, the variation of SE is not dependent on the type of signal, but on the self-order of the frequency content of the seismic signal prior to eruptive processes. Our hypothesis is the majority of the elastic energy recorded in the seismogram is associated with this eruptive process and must be similar to itself. Otherwise, when there is no imminent eruptive process, the volcano can show different seismic signals that do not reflect homogeneity of the seismogram, and the values of SE are higher.

We developed a numerical rule to quantify when the SE begins to decrease in a regular value approaching zero. Through this type of measure, we will establish an interval to determine when the volcanic system is in a pre-eruptive process. Rey-Devesa et al.^29^ showed the accuracy of the LTA/STA algorithm to implement this quantification. Following these authors, we define the LTA value by calculating the mean value of the SE using a period of two months previous to the STA window, ${\mathrm{SE}}_{0}$. Then, we compare this value with the SE value in each window of the analysis, $\mathrm{SE}(\mathrm{i})$, estimating a Decay Ratio using Eq. (2):

2 $$Decay\, Ratio\, [\%]=100\cdot \left(1-\frac{SE(i)}{{SE}_{0}}\right)$$

A decay of the STA/LTA values over the 70% could be considered as indicator of potential eruptive episodes, avoiding potential false eruption alarms^29^. Systematically we computed the SE decay (Eq. 2) obtaining its temporary evolution. Then, we identified those intervals where there are decays of the SE below the threshold and analysed the images recorded by the CUEV cameras system to confirm whether there is an eruptive event. In case of positive confirmation, we evaluated the length of the pre-eruptive interval of each eruptive event.

We apply advanced signal analysis techniques to use the SE as a short-term volcanic eruption forecasting tool for three purposes:

- To measure SE temporal evolution prior to a set of energetic eruptions, determining the time interval to alert the population and prepare for volcanic hazard.
- To test if SE is able to distinguish between high and low energy episodes and determine if the pre-eruptive interval is associated to the energy of the explosions.
- To introduce SE to redefine the label associated with eruptions in classifications models.

## Results and discussion

### Three years of seismic record

**Figure 2:**
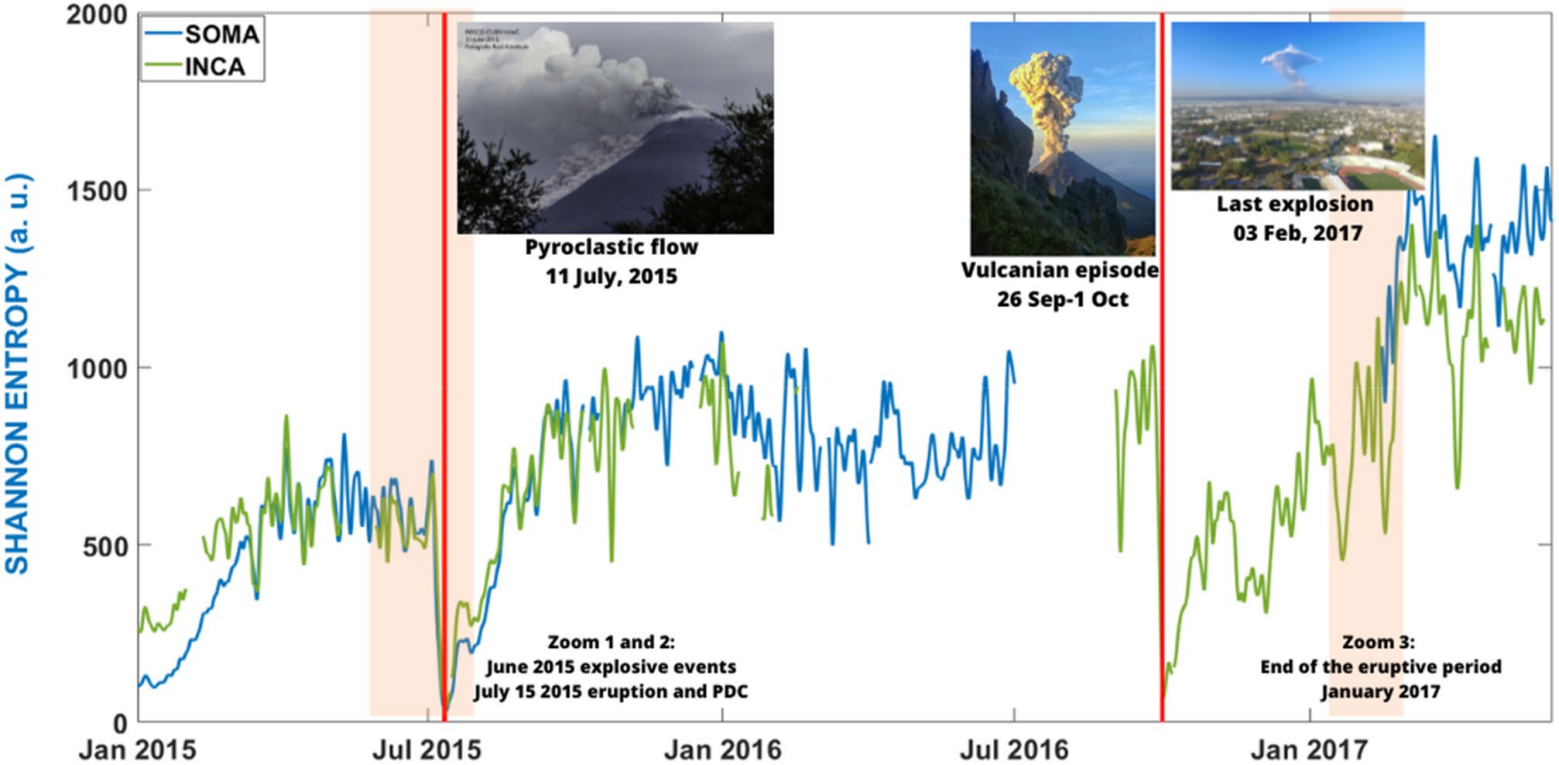
Temporal evolution of the SE between January 2015 and May 2017 analysed at SOMA (blue) and INCA (green) seismic stations using window lengths of 10 min overlaped 50%. We represented the envelope of the SE values. Vertical red line represents the two selected eruptive episodes occurred on July 11th 2015 and October 1st 2016. Shadow red areas represent the two intervals selected to analyzed smaller volcanic explosions. White spaces represent periods without data. Pictures from left to right show three explosive episodes recorded by the CUEV cameras occurred on 11 July 2015, 1 October 2016 and 3 February 2017 respectively.

The first step is to evaluate the robustness of the temporal evolution of the SE. We selected the eruptive stage occurring on Volcán de Colima between January 2015 and May 2017, analysing more than two years of data recorded at 4 different seismic stations. In Fig. 2 we show the envelope of the SE evolution, calculated with the signal recorded at stations SOMA (blue) and INCA (green), selected for being near the crater (less than 2 km far) and for their complementary records throughout time. The temporal evolution of the SE plotted in Fig. 2 starts from low values. The 28 of December 2014 Volcán de Colima had an intense eruptive episode dominated by lava flows (Carrara et al., 2019). This eruptive episode was not included in our analysis since we focused our study in explosive episodes. SE is sensible to any type of eruptive mechanism ^29^**.** It is also interesting to observe how after the last volcanic explosion and the beginning of a rest period of Volcán de Colima, the SE has higher and more stable values. Finally, we highlight that the two main volcanic processes selected (11 July 2015 and 1 October 2016) show how SE reaches values very close to zero evidencing their high energy and the coherence of the seismic process prior to the eruptions.

### Shannon entropy as a precursor

#### The 11th of July 2015 volcanic explosion

**Figure 3:**
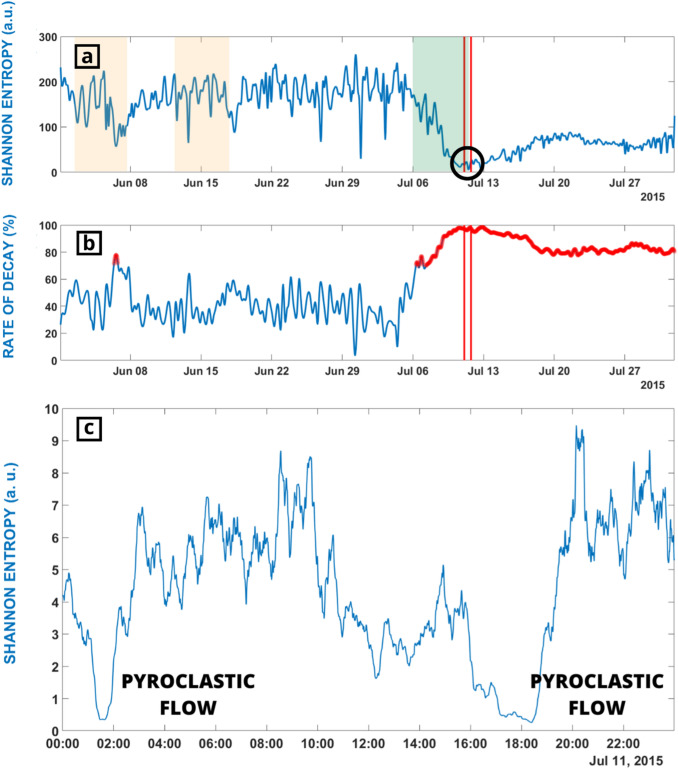
(**a**) SE during June and July 2015. Red lines show the moment of the two pyroclastic flows occurred in July 11th. Red shadow areas are the periods used to evaluate how SE can be used to monitor volcanic explosions. Green area is the confirmed short term forecasting period (5 days) obtained from the decay of the SE. (**b**) Plot of the STA/LTA ratio during June and July 2015. Period in which values are over 70% of decay are highlighted in red. (**c**) Zoom of 11 of July showing as the SE reached zero when the pyroclastic flows happened.

Prior to the high energy volcanic explosion of July 11th, SE trend dropped to minimum values close to zero (Fig. 3a). According to Fig. 3a,b the pre-eruptive short-term interval of this explosion was of 5 days (green area of Fig. 3a and red line of Fig. 3b). This interval corresponds to the stable decay of the SE below the 70% of threshold. Notice that when the two associated pyroclastic flows happened the SE has a decay value of 100%. Fitting the decay of the SE we could be able to determine in advance the timing of the first pyroclastic flow (Fig. 3c).

This explosion presented a low VT or LP level of seismicity on the base of the use of ML techniques as Hidden Markov Models^54^ lacking classical pre-eruptive precursors^30,48^.

#### The 1st of October 2016 volcanic eruption

The selected eruptive interval started with an effusive style finalizing with a Vulcanian explosion. The decay of the SE to values close to zero occurred just before the vulcanian explosion (Fig. 2). Again, non-intense pre-eruptive VT or LP seismicity was reported^55^. However, the SE shows a pre-eruptive decay two days in advance. Notice as the pre-eruptive time is shorter than in the previous explosion that was more energetic than the present one (Fig. 2). We realized the pre-eruptive interval determined by the decay of SE seems to be associated to the magnitude of the eruptive episode^29^ (Supplementry Fig. S1).

### Tracking volcanic explosions

#### The intense explosive period of June 2015

The most leading precursory activity of 11 of July 2015 pyroclastic flows was the high number of small volcanic explosions occurred in June^48^. This type of eruptive activity is characteristic of Volcán de Colima and it is composed by small vulcanian and hydrothermal explosive activity. The hydrothermal activity use to have a shallower source in comparison to the vulcanian ones^39,45^. This shallower character also conditions the seismic feature, in general showing less energetic seismic signals and also strongly affected by attenuation effects. There are examples in which phreatic and hydrothermal explosions are very energetic producing large effect, including causalities, as was the case of White Island in New Zealand^17,56,57^. However, the deep hydrothermal interaction on Volcán de Colima has different character. The deep aquifer and the deep hydrothermal system below Volcán de Colima could modify the eruptive style, including the occurrence of lateral multi-stage volcanic edifice as El Volcancito, but not generating large phreatic explosions^35^.

**Figure 4:**
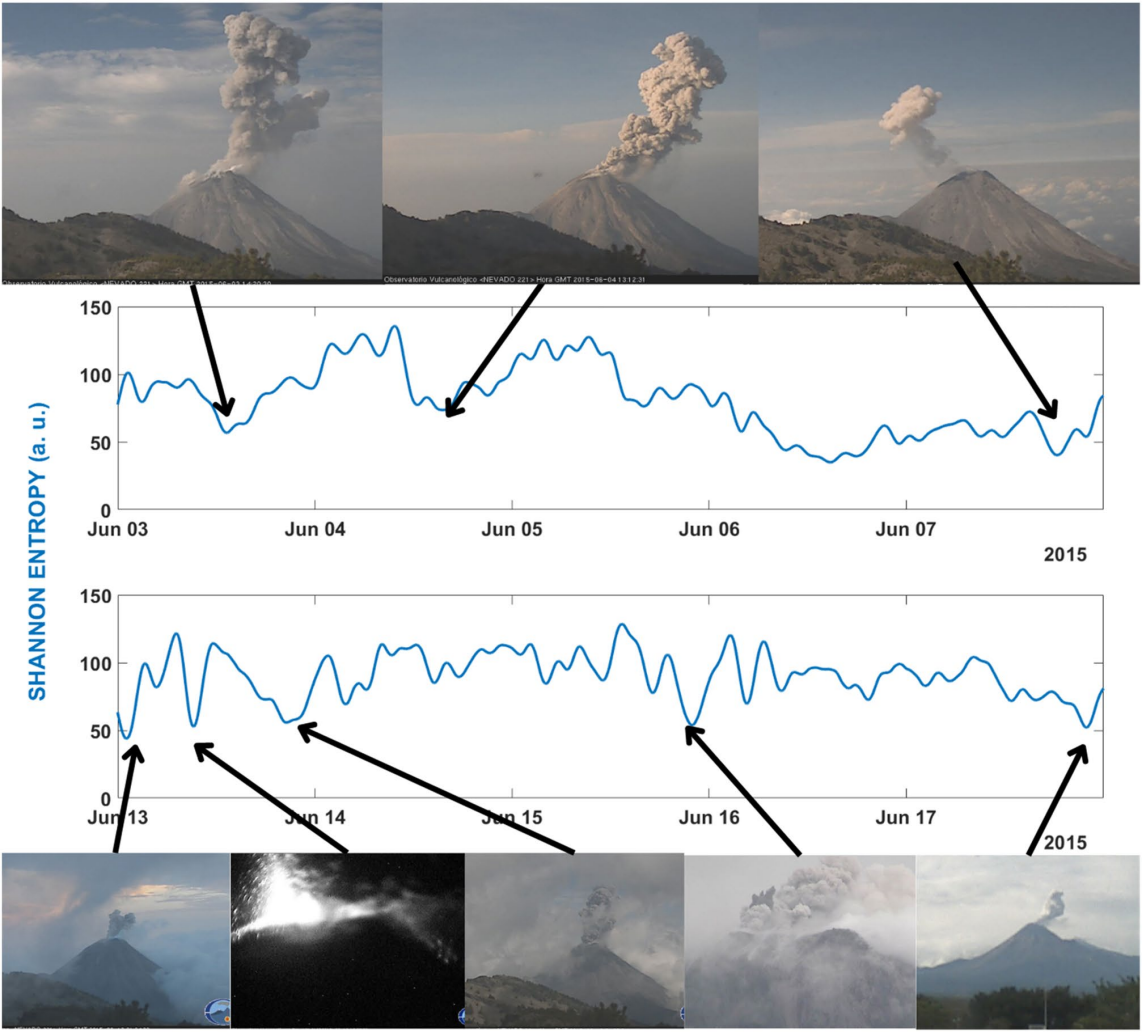
Temporal evolution of the SE values, obtained for the seismic station SOMA, associated to the high explosivity period of 3–20 June 2015. In this period, we identified local minima and compared them (if available) with the images obtained by the visual monitoring Network. As observed all identified minima are linked low energy explosive activity.

In Fig. 4 we zoomed in detail this period in order to observe the temporal evolution of SE according to the less energetic explosions. This study corresponds to a re-analysis of this period using windows 1 min long overlapped the 50%. Doing a manual blind test, we identified several excursions of SE towards lower values (local minima). Then, we associated them with the corresponding images recorded by the visual monitoring system. As observed in Fig. 4, most of the local minima of the SE were associated to small volcanic explosions, whenever the weather conditions allowed getting these images. This double study is not always easy to perform. It is a fact that in many cases the presence of clouds or night does not facilitate this work. These variations towards the local minima take place in a short time and it is not possible to assign a potential forecasting interval, as observed in the two largest eruptive episodes analysed before; thus, the local minima associated to small volcanic explosions do not always are below the STA/LTA threshold established for volcanic early warning. The main goal of this second analysis is the development of a tool capable of tracking these explosions and improve the seismic and volcanic catalogues.

#### The explosive sequence of January 2017 prior to the quiescence volcanic stage

After February 2017, the eruptive activity of Volcán de Colima ceased^58^. This is reflected in Fig. 2, where we can appreciate how SE started to grow reaching the maximum values of all the period studied between March and May 2017. We finalized our study analysing how SE evolves during the end of an eruptive period. Ten volcanic explosions have been identified between January 7th and February 3rd, 2017, prior to the quiescence phase started after them (end of February 2017)^58^.

**Figure 5:**
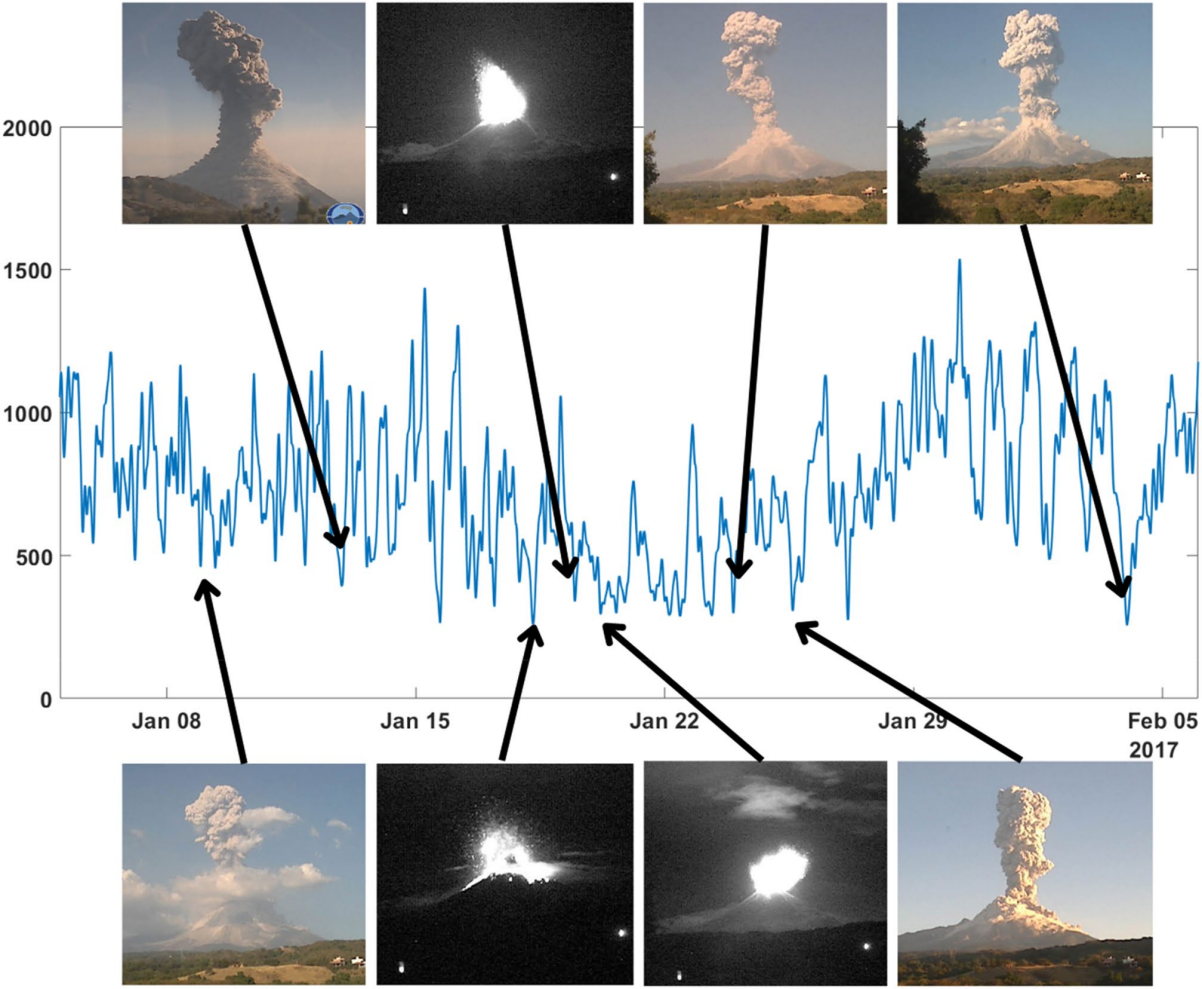
Temporal evolution of the SE values, obtained for the seismic station INCA, associated to period of 3 January-5 February 2015. In this with period we identified local minima and compared them (if available) with the images obtained by the visual monitoring Network. As observed all identified minima are linked to explosive activity.

As observed in Fig. 2, even if there are several volcanic explosions in the period selected in this study, the SE was in a trend to have higher values than in previous months. We can interpret this increase of the SE values due to the approaching of the end of the eruptive episode. However, we could identify relative minima of the SE and associated them with images of the visual monitoring network. In Fig. 5 we associated the pictures of 8 of the 10 explosions reported by Arámbula-Mendoza et al.^58^ with the minima of the SE. The other 2 explosions (January 7th and 27th) were not recorded on camera due to high fog, but we can observe minima values for SE (Fig. 5).

##### ***Remarks.***

We remark the systematic analysis of the SE can be a useful tool in the processes of monitoring and seismic surveillance of volcanoes. Analysing three years of seismic signals at Volcán de Colima SE presents high and stable values when the volcanic activity is low or the volcano is quiescent, while whenever the SE has local minima, or tends towards values close to zero, it is marking eruptive processes (Supplementry Fig. S2).

SE measures the uncertainty in probability distributions^59,60^, associating maximum SE with maximum uncertainty (all possible outcomes have equal probabilities), and vice-versa coherent outcomes show high probabilities (minimum SE). On the other hand, the Entropy defined by the Statistical Physics establishes that the macroscopic state of a physical system is characterized by a distribution of microstates^61^. A volcanic system is a set of different inner processes defined by a set of microstates defining the exchange of energy with the medium. The configuration of equilibrium of a volcanic system, for example a quiescence period, is associated with minimum exchange of energy and low values of Entropy. Seismic record associated to this state is characterized by a random low energetic composition of signals providing high SE values. In opposition, seismic signals with similar temporal and frequency patterns (same source) provide low SE values. Spectral sorting of a system is indicated by the lowering of SE values, as van Ruitenbeek et al.^62^ studied for hydrothermal systems. In tectonic seismology high Entropy and low SE are associated to the arrival of large earthquakes with impulsive and energetic phases generated in the same source**.** In volcanic systems, the increase of the Entropy is related to several microstates associated to the inner dynamic of the volcano. Particles of gas, magma, bubbles, solid material and other components existing in the interior of the volcano interact between them and with the limits of the volcanic structure, exchanging energy. When these microstates are coherently organized to generate a “volcanic macrostate”, i.e., oriented to produce a volcanic eruption, then the values of the SE are moved towards zero and the Entropy is maximum. This behaviour is independent of the nature of the pre-eruptive seismicity, as it is observed in Bezymianny volcano, dominated by VT seismic swarm, Mt. Etna, by volcanic tremor and Mount- St. Helens, by mixed VT and tremor^29^.

## Conclusion

This study shows that SE could be a useful tool for volcano monitoring and provide in many cases evidences to be used as short-term volcanic eruption early warnings. The temporal analysis of SE shows interesting behaviour whenever the volcanic activity changes to an eruptive state, in both cases for high and low energetic episodes. The volcanic system self-organises prior to an eruption. This self-organization is reflected through a homogeneous composition of the seismic signal. We can interpret this self-similarity as a way out of the trend that the volcanic activity was following, reflected as minimum values of SE. Moreover, they offer new information about the eruptive state of the volcano. Thus, when SE moves toward zero the most probable interpretation of this variation is an energetic eruptive episode of the volcano. This study makes an approach to a better understanding of the activity and the processes underlying in a volcanic system close to an eruption. Thus, when a high energy explosion is approaching, SE starts to decrease from days before in a homogeneous way until reaching absolute minimums when the volcanic eruption happens. Furthermore, we have observed that SE is as a reliable feature for the improvement of ML automatic classification systems and the identification of low energy explosions. To improve the training of ML approaches with additional data of volcanic eruptions is crucial.

Finally, in the case of Volcán de Colima we demonstrated the two big eruptions selected could be forecasted with a few days in advance (6 and 2 days respectively) using the homogeneous decay of the SE. We showed SE was sensible to another previous eruptive episode, occurred at the end of 2014 (very low SE values), and also to the end of the eruptive stage and beginning of a quiescence period (high and stable SE values).

We conclude that SE could be used as a complementary tool in seismic volcano monitoring. The SE has coherent decreasing behaviour prior to energetic eruptions, giving time enough to alert the population and prepare for the consequences of an imminent and well predicted moment of the eruption.

## Supplementary Information

Supplementary Information.

## Data Availability

The used software of this work (all programmes developed by us) are also publicly accessible (link provided below) and are also presented with specific use examples to be able to independently reproduce all the results obtained in this work. The repository sites used are stable, publicly accessible for free and recognized by the scientific community. The seismic parameter analysis software, with illustrative examples to be able to reproduce our results, are available in the compressed folder “Software.Rar”, located in the ZENODO repository at the following address and https://doi.org/10.5281/zenodo.6821530 . https://zenodo.org/record/6821530#.YvyeUS7P1PY. The seismic data used in this work are accessible in: https://doi.org/10.5281/zenodo.7732898.
