# Supplementary material for: Tracking volcanic explosions using Shannon entropy at Volcán de Colima
Source: Sci Rep. 2023 Jun 17;13:9807. doi: 10.1038/s41598-023-36964-x (PMC10276871; doi:10.1038/s41598-023-36964-x)
Supplement: Supplementary file 1 — Supplementary Information. [file 41598_2023_36964_MOESM1_ESM.pdf]

Tracking Volcanic Explosions using Shannon Entropy at Volcán de Colima.

Supplementary Material

Pablo Rey-Devesa[1,2,*], Janire Prudencio[1,2], Carmen Benítez[3], Mauricio Bretón[4], Imelda Plasencia[4], Zoraida León[4], Félix Ortigosa[4], Ligdamis Gutiérrez[1,2], Raúl Arámbula-Mendoza[4] & Jesús M. Ibáñez[1,2].

[1]Department of Theoretical Physics and Cosmos. Science Faculty. Avd. Fuentenueva s/n. University of Granada. 18071. Granada. Spain.

[2]Andalusian Institute of Geophysics. Campus de Cartuja. University of Granada. C/Profesor Clavera 12. 18071. Granada. Spain.

[3]Department of Signal Theory, Telematics and Communication. University of Granada. Informatics and Telecommunication School. 18071. Granada. Spain.

[4]Centro Universitario de Estudios Vulcanológicos (CUEV), Observatorio Vulcanológico, Universidad de Colima, Colima, México.

*Corresponding author: pablord@ugr.es

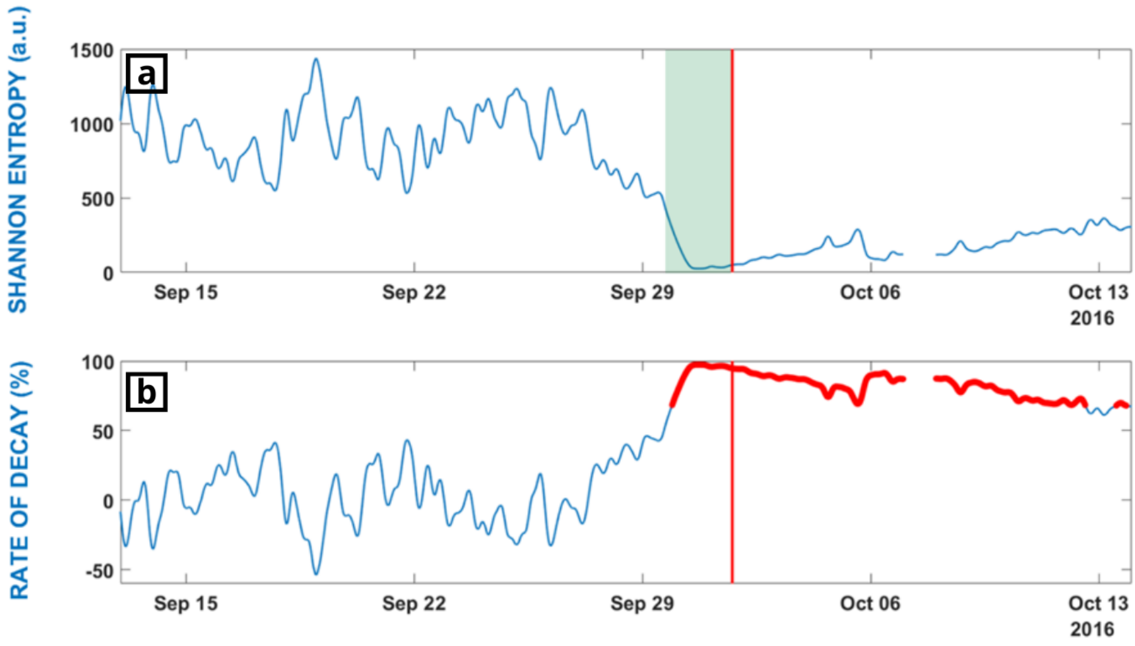

Figure S1: a) *SE during September and October 2016. Red line shows the moment of the vulcanian eruption occurred in October 1st. Green area is the confirmed short term forecasting period (2 days) obtained from the decay of the SE.* ***b)*** *Plot of the STA/LTA ratio during June and July 2015. Period in which values are over 70% of decay are highlighted in red.*

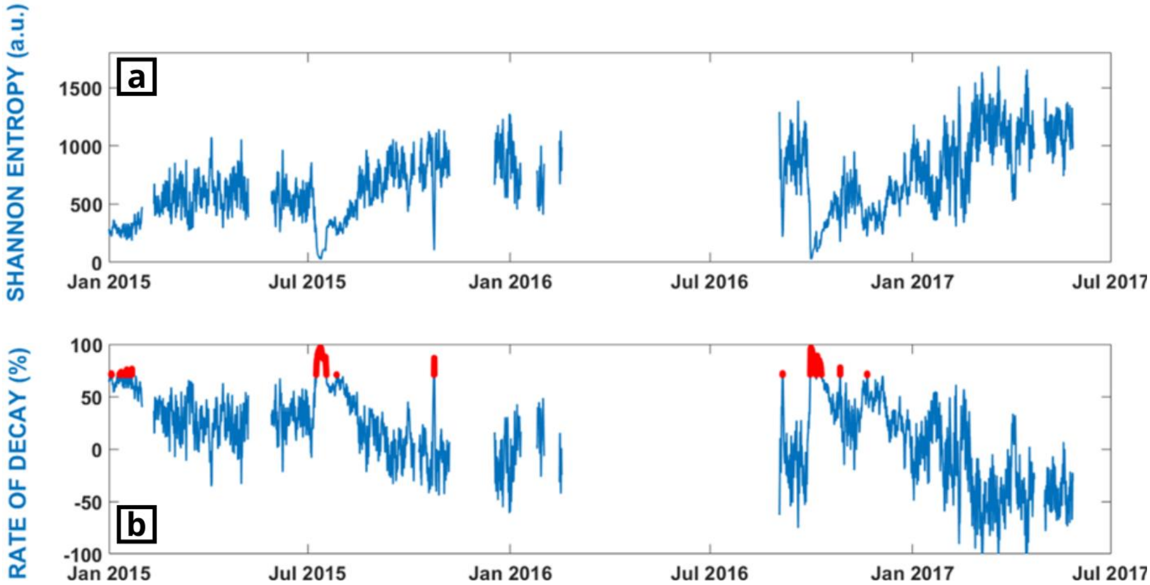

Figure S2: a) *SE during the period analyzed. Seismic station INCA.* ***b)*** *Plot of the STA/LTA ratio. Period in which values are over 70% of decay are highlighted in red. Notice that the highlighted regions correspond to the eruptive period in the end of 2014, the eruption in July 2015, and the eruption in October 2016. The spikes highlighted on October 23rd 2015 and September 4th 2016 are associated to the arrival of the hurricanes Patricia and Newton respectively [1,2].*

References:

1. Walter, T. R., Salzer, J., Varley, N., Navarro, C., Arámbula-Mendoza, R., & Vargas-Bracamontes, D. (2018). Localized and distributed erosion triggered by the 2015 Hurricane Patricia investigated by repeated drone surveys and time lapse cameras at Volcán de Colima, Mexico. Geomorphology, 319, 186-198.
2. Zobin, V. (2022). Dynamics of Hurricanes and Tropical Storms along the Pacific Coast of Mexico: A View from a Seismic Station. Prevention and Treatment of Natural Disasters, 1(2).
